# Effect of piracetam on the cognitive performance of patients undergoing coronary bypass surgery: A meta-analysis

**DOI:** 10.3892/etm.2013.1425

**Published:** 2013-11-26

**Authors:** YU FANG, ZHANDONG QIU, WENTAO HU, JIA YANG, XIYAN YI, LIANGJIANG HUANG, SUMING ZHANG

**Affiliations:** Department of Neurology, Tongji Hospital of Tongji Medical College, Huazhong University of Science and Technology, Wuhan, Hubei 430030, P.R. China

**Keywords:** piracetam, bypass coronary surgery, cognition disorders, neuroprotective agents, meta-analysis

## Abstract

Cognitive impairments are observed in numerous patients following coronary bypass surgery, and piracetam are nootropic compounds that modulate cerebral functions by directly enhancing cognitive processes. The present meta-analysis was conducted to evaluate the protective effect of piracetam on the cognitive performance of patients undergoing coronary bypass surgery. The relevant studies were identified by searching Medline, EMBASE, PubMed and the Cochrane Library up to June 2013 and the pertinent bibliographies from the retrieved studies were reviewed. Data were selected from the studies according to predefined criteria. The meta-analysis included two randomized control trials involving 184 patients and including the Syndrom-Kurz test (SKT). Findings of the meta-analysis showed that following treatment the change from baseline observed in five SKT subtest scores, conducted with piracetam patients, indicated a significant advantage over those patients that were in the placebo group. The subtests included immediate pictured object recall, weighted mean difference (WMD)=0.91, 95% confidence interval (CI) 0.51–1.31, P<0.00001; delayed pictured object recall, WMD=0.74, 95% CI 0.19–1.28, P=0.008; delayed picture recognition, WMD=0.82, 95% CI 0.31–1.31, P=0.001; immediate word recall, WMD=0.87, 95% CI 0.47–1.28, P<0.0001; and letter interference, WMD=3.46, 95% CI −5.69 to −1.23, P=0.002. These results indicated that piracetam may have been effective in improving the short-term cognitive performance of patients undergoing coronary bypass surgery. High quality, well-controlled and longer randomized trials are required to corroborate this result.

## Introduction

Cardiovascular disease is the predominant cause of mortality in America, claiming ~420,000 lives per year or approximately one fatality per minute (1). Coronary artery bypass grafting (CABG) surgery is a common cardiac intervention recognized as an effective method of stabilizing ventricular dysfunction (2). The majority of patients undergoing CABG surgery receive a cardiopulmonary bypass (CPB) to maintain the circulation of blood and the oxygen content in the body. However, a significant amount of patients that undergo CABG surgery develop postoperative cognitive dysfunction (POCD) (3–5). Previous studies have indicated that the incidence of POCD varies between 25 and 80% (2), ranging from slight to pronounced disturbances that may persist for several weeks or up to several years (3,6–9). These cognitive impairments may result in increased in-hospital mortality, prolonged hospitalization and increased use of health service resources. Furthermore, as these cerebral disturbances vary in extent and duration, they may reduce the patient’s quality of life for a prolonged period of time (10).

The exact pathophysiological mechanisms for the CABG-associated cognitive decline remain unclear. The reason for this may result from a direct toxic influence of narcotics and/or a slight to marked hypoxic condition that is experienced during anesthesia. An additional, significant reason may be stress resulting from the CPB. Previous studies have reported that cognitive decline is multifactorial (3,6,9), including factors such as microemboli (11,12), ischemic hypoperfusion brain lesions (13), hemodilution (14), low body temperature during surgery (15), individual susceptibility to cerebrovascular disease (16), inflammatory responses (17) and possession of the gene for apolipoprotein Eɛ4 isoform in addition to microemboli (18). Specific studies have been conducted to investigate treatment methods that are based on the potential mechanism of the impairment and certain agents have been tested regarding POCD. Heparin (19), lidocaine (20), and piracetam (21) were identified as beneficial, whereas the positive effects of prostacyclin (22), GM1 gangliosides (23), remacemide (24), pexelizumab (25) and S-(+)-ketamine (26) have yet to be confirmed. Regardless of numerous recent attempts, no gold standard method has been devised for the treatment of cognitive impairments that are associated with CABG surgery.

Among the potential drugs mentioned above, piracetam belongs to a class of nootropic compounds, which modulate cerebral function by directly enhancing cognitive processes, such as learning, memory, attention and consciousness (27). The cognitive-enhancing properties of piracetam have been previously demonstrated (28,29): Piracetam is administered to restore cognitive performance in patients with encephalopathy of various etiologies, including cranial trauma, inflammation, strokes and ischemic complications following coronary bypass surgery (30). Furthermore, no severe adverse effects of piracetam have been indicated. In a previous study, Richardson and Bereen (31) demonstrated that a significantly higher percentage of patients receiving piracetam attained or maintained a normal or near-normal level of consciousness postoperatively, when compared with those that received a placebo. Saletu *et al*(32) showed that subjects that were treated with piracetam performed better than those in the control group. A series of clinical studies indicated that the perioperative administration of nootropic piracetam resulted in a reduced recovery period following anesthesia and improved the symptoms of delirium (33). To analyze the potential therapeutic properties, certain double-blind, placebo-controlled clinical trials were designed to evaluate the effect of piracetam in preventing cognitive impairment following CABG surgery. The ability of piracetam to limit the extent of cognitive impairment following coronary bypass surgery remains unknown. Therefore, a meta-analysis was conducted to evaluate the effectiveness of piracetam on the cognitive performance observed in patients who have undergone coronary bypass surgery.

## Materials and methods

### Search strategy

Two independent computer-assisted searches were conducted using the Medline, EMBASE, PubMed databases and the Cochrane Library to obtain literature up to June 2013. The search was performed using a combination of the keywords ‘piracetam’ and/or ‘cardiac surgery’, ‘coronary artery bypass graft’, ‘heart surgery’, or ‘thoracic surgery’; no limits were imposed based on language. The titles and the abstracts of the identified studies were separately assessed to confirm fulfillment of the inclusion criteria, while any disagreements were resolved through consultation. The data extraction was performed independently using standard data extraction forms and the quality of the identified studies was assessed in terms of the randomization method, the allocation concealment, the blinding method (participants, investigators, outcome assessors and data analysts) and the completeness of the follow-up.

### Inclusion criteria

Studies were included depending on the following criteria; i) the design was a randomized controlled trial (RCT), ii) the population comprised participants undergoing coronary bypass surgery, iii) one of the interventions was treatment with piracetam, and iv) the study included outcome measures of cognitive function. Following selection based on the title and abstract, the full-text studies were retrieved. Reviews, case reports and conference proceedings were excluded. The methodological quality of the RCTs was assessed using a modified augmented Jadad scale (34), which accounts for whether the study describes randomization, blinding and withdrawals/dropouts to provide a quality score out of 10. Total scores >5 were included in the present meta-analysis.

### Data selection and calculation of the effect size

The effect sizes were reported in all of the placebo-controlled studies where the data values were available. The outcome and date were obtained from the studies and the information was selected to record the sample characteristics, the independent variable, the outcome variable, the results and the statistical methods. Calculations were performed to obtain the average difference between the piracetam and placebo treatment groups for the change in cognitive function test scores over the course of the trial, from each cognitive function test that was used per trial. The effect sizes were computed for each study, using the mean difference (MD) of the change in score from the baseline (MD_change_) and the standard deviation of the change from the baseline (SD_change_). When the MD_change_ and/or SD_change_ was not provided in the study, the change of the parameter was calculated using the formula (35):

$$\begin{array}{l} {\text{MD}}_{\text{change}}={\text{M}}_{\text{final}}-{\text{M}}_{\text{baseline}}\\ {\text{SD}}_{\text{change}}=\surd ({{\text{SD}}^{2}}_{\text{final}}+{{\text{SD}}^{2}}_{\text{baseline}}-{\text{SD}}_{\text{final}}\times {\text{SD}}_{\text{baseline}}) \end{array}$$

where M_final_ is the MD of the final score, M_baseline_ is the MD of the baseline score, SD_final_ is the standard deviation of the final score, and SD_baseline_ is the SD of the baseline score.

The meta-analysis of the global outcome was conducted subsequent to the development of the methodology set out by the Cochrane Database of Systematic Reviews (35). For the continuous variables, the weighted mean difference (WMD) and corresponding 95% confidence intervals (CI) were computed using the random-effects model. The heterogeneity was estimated using I^2^ statistic for RCTs. The analysis was conducted using Review Manager 5.0 software (Cochrane IMS; http://www.cochrane.org/editorial-and-publishing-policy-resource/review-manager-revman). The I^2^ values were calculated and described the heterogeneity between the results obtained from the studies. The thresholds for the interpretation of I^2^ were based on previous studies, indicating that 0–50, 51–75 and 76–100% represents mild, moderate and considerable heterogeneity, respectively. P<0.05 was considered to indicate a statistically significant response.

## Results

### Search results

Six potentially eligible trials were identified, however, three trials were subsequently excluded. The three trials that were selected included varying treatment doses and durations (Table I). Two RCTs comprising 184 patients were included for the meta-analysis and six SKT subtest scores were selected to evaluate the cognitive function of the patients one day prior to surgery and on the third postoperative day. The six subtest of SKT scores included immediate pictured object recall, immediate word recall, attention, letter interference, delayed pictured object recall and delayed picture recognition. The six subtest scores from the studies were compared (21,36). Szalma *et al*(37) reported that cognition was observed to be significantly improved in those patients that were treated with piracetam for six weeks following CABG surgery. However, these data were not included in the meta-analysis due to the different doses and durations of the medical treatment. No unpublished literature was identified during the search conducted for the present analysis and among the studies that were included, the occurrence of adverse events was rare (Table I).

### Meta-analysis results

**Figure 1:**
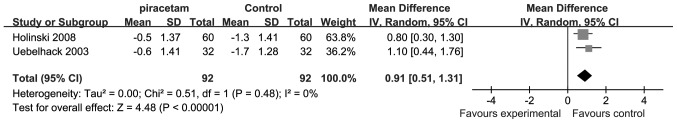
Meta-analysis variations from the baseline following treatment in the piracetam group versus the control group, relating to the immediate pictured object recall test. P<0.05 was considered statistically significant. SD, standard deviation; IV, inverse variance; CI, confidence interval.

**Figure 2:**
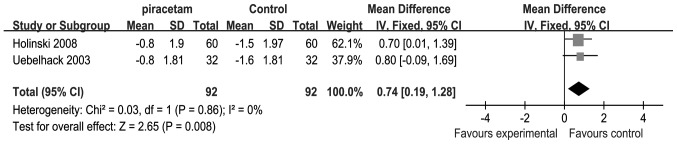
Meta-analysis variations from the baseline following treatment in the piracetam group versus the control group, relating to the delayed pictured object recall test. P<0.05 was considered statistically significant. SD, standard deviation; IV, inverse variance; CI, confidence interval.

**Figure 3:**
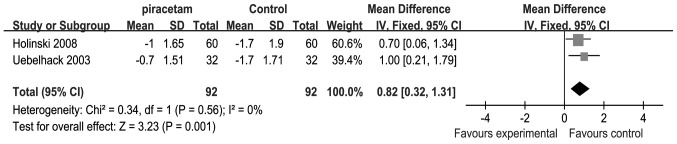
Meta-analysis change from the baseline following treatment in the piracetam group versus the control group, relating to the delayed picture recognition test. P<0.05 was considered statistically significant. SD, standard deviation; IV, inverse variance; CI, confidence interval.

**Figure 4:**
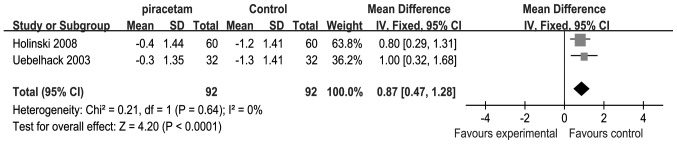
Meta-analysis variations from the baseline following treatment in the piracetam group versus the control group, relating to the immediate word recall test. P<0.05 was considered statistically significant. SD, standard deviation; IV, inverse variance; CI, confidence interval.

**Figure 5:**
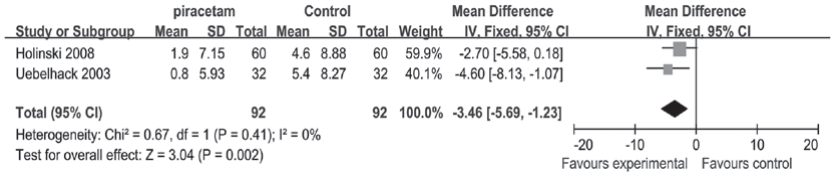
Meta-analysis variations from the baseline following treatment in the piracetam group versus the control group, relating to the letter interference test. P<0.05 was considered statistically significant. SD, standard deviation; IV, inverse variance; CI, confidence interval.

**Figure 6:**
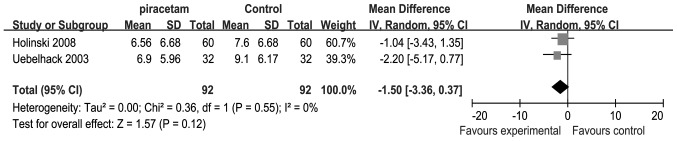
Meta-analysis variations from the baseline following treatment in the piracetam group versus the control group, relating to attention. P<0.05 was considered statistically significant. SD, standard deviation; IV, inverse variance; CI, confidence interval.

The findings are reported in terms of WMD, 95% CI and a P-value for each subtest for the overall effect. A statistically significant difference was identified between the piracetam and control groups in the change from the baseline in five of the subtest scores (immediate pictured object recall, WMD=0.91, 95% CI 0.51–1.31, P<0.00001 (Fig. 1); delayed pictured object recall, WMD=0.74, 95% CI 0.19–1.28, P=0.008 (Fig. 2); delayed picture recognition, WMD=0.82, 95% CI 0.31–1.31, P=0.001 (Fig. 3); immediate word recall, WMD=0.87, 95% CI 0.47–1.28, P<0.0001 (Fig. 4); letter interference, WMD=−3.46, 95% CI −5.69 to −1.23, P=0.002 (Fig. 5). Furthermore, there was no evidence of heterogeneity identified between the studies (I^2^=0%). Moreover, no statistically significant difference was indicated between the piracetam and the control therapy groups in the change from baseline in the attention scores (WMD=−1.50, 95% CI −3.36 to 0.37, P=0.12) with no heterogeneity observed between the three studies (I^2^=0%; Figs. 1–6).

## Discussion

The meta-analysis of 184 patients undergoing coronary bypass surgery, which were randomized to piracetam or control therapy groups, indicated that there was a statistically significant difference observed in the change from baseline in the five SKT subtest scores, including immediate pictured object recall, immediate word recall, letter interference, delayed pictured object recall and delayed picture recognition, however, no difference was observed in the attention scores. The six parameters that were used to detect the cognitive function were selected from the repeatable battery of SKT subtests, which is a standardized screening instrument that was designed to assess cognitive function over a brief administration period (38). The results indicated that piracetam may benefit early cognitive function for patients that have undergone coronary bypass surgery, although it may be less effective in improving the attention scores following coronary bypass surgery. However, the present analysis was limited by the small number of patients and the pharmacogenomic variations that may exist between the different populations. In the research design, the patients that exhibited the following diseases were excluded from the present study: insulin-dependent diabetes mellitus, renal insufficiency that required dialysis and/or a history of transient ischemic attack, prolonged reversible ischemic neurologic deficit, or a complete stroke. However, elderly patients requiring CABG surgery are often associated with other diseases, particularly cerebrovascular accidents and diabetes. It remains unknown whether piracetam may benefit these patients, however, it is significant for clinical treatment. Therefore, it is necessary to focus on these factors to enable the use of this drug as a routine treatment.

The underlying pathophysiology of POCD following CPB remains unclear (39). Regardless of the declining incidence of other complications, the reported incidence of postoperative cognitive complications remains predominantly unchanged (40). Cognitive deficits following cardiac surgery were considered to be a result of physiological disturbances, which were associated with the cardiopulmonary bypass technique and there is a lack of studies relating to the treatment of POCD following CPB. Non-pharmacological and pharmacological strategies have been shown to potentially prevent the CPB-associated cognitive decline with one study indicating that patients may benefit from a cognitive training program, which is designed to improve performance in attention and memory tasks (41). Previous studies demonstrated that intraoperative neuromonitoring aids with the prevention of POCD, reduces the duration of the hospital stay and the cost, and minimizes the adverse effects observed in several vital organs (42,43). Moreover, the results of a previous study indicated that gastrodin is an effective and safe drug for the prevention of neurocognitive decline in patients that undergo mitral valve replacement surgery with a CPB (44). However, regardless of these benefits, there is currently no standardized neuroprotective drug available to patients that undergo CPB surgery.

Piracetam, the prototype of the nootropic drug, is administered in numerous countries for the treatment of cognitive impairment resulting from aging, brain injuries and dementia (29). Piracetam affects neuronal metabolism, improves the utilization of glucose within neurons and improves the synthesis of neuromediators and nucleic acids within the brain. These mechanisms may be significant in the prevention of POCD following general anesthesia. Furthermore, piracetam has been shown to alter the physical properties of the plasma membrane by increasing its fluidity and protecting the cell against hypoxia (45) by increasing the red blood cell deformability and normalizes the aggregation of hyperactive platelets, which may regulate the formation of microemboli. Thus, with antithrombotic, neuroprotective and rheological properties, piracetam may contribute to the improvement of the cognitive impairment resulting from CPB surgery.

However, there were certain limitations in the present meta-analysis, including that the study population may not be representative of the average patient population that undergoes CABG surgery, which may result in a public bias and thus a low CI. Therefore, these results must be treated with caution and large sample, well-designed trials are required to confirm these results. Future investigations should focus on the impact of geographical variations and consider whether the patients exhibit other underlying diseases. Moreover, no universal analytical criteria exist for POCD and the heterogeneity limits the comparisons of POCD that can be performed between studies. Thus, a unified battery and specific analytical criteria may improve the comparability and address the challenge of measuring the process of learning, the floor and ceiling effects, and may ultimately advance this scientific field. Furthermore, it may result in an improved understanding of the reasons for POCD and, therefore, develop strategies for its prevention or treatment. Moreover, no uniform criteria exist to assess the cognitive deficits resulting from surgery; thus, the availability of specific and sensitive measures of cognitive function may enhance the detection of cognitive deficits following CABG surgery.

Therefore, further investigations are required to understand the molecular mechanisms of the antithrombotic, neuroprotective and rheological properties, which are believed to account for the effects of piracetam. In clinical research, further studies are required to evaluate the duration and dosage of piracetam that should be administered to achieve a satisfactory treatment outcome. Recent studies have exclusively investigated the short-term effect of piracetam on cognitive function, however, future investigations are necessary to determine the long-term effects. The selection of a suitable or standard set of analysis criterion may be advantageous in future studies.

In conclusion, the results of the present study indicated that piracetam may have been effective in the improvement of short-term cognitive performance in patients that have undergone CPB surgery. However, confirmation of the results, by well-designed trials with large samples, are required.

## Figures and Tables

**Table I:** Identified studies on the administration of piracetam in the perioperative period and the effect on cognitive performance.

Author (Year)	Study design	No. of subjects (active, placebo)	Piracetam dose	Duration	Age, years (SD)	Outcomes used for meta-analysis	Main conclusion	Jadad score
Uebelhack (2003)	RCT	(32, 32)	12 g/60 ml IV over 30 mins	Once prior to surgery	63.1 (8.4)	SKT	Piracetam infusion prior to surgical intervention may provide a short-term neuroprotective effect	7
Szalma (2006)	RCT	(50, 48)	Prior to surgery: 150 mg/kg/day IV Post surgery: 12 g/day PO	Six weeks	P: 55.50 (5.58) C: 56.16 (5.51)	Twelve neuro-psychologic tests	Six weeks after CABG cognition was significantly improved in patients treated with piracetam	7
Holinski (2008)	RCT	(60, 60)	12 g/60 ml IV over 30 mins	Once prior to surgery	62.2 (8.5)	SKT	Piracetam reduces early postoperative substantial decline of neuro-psychological abilities	7

SD, standard deviation; RCT, randomized controlled trial; IV, intravenously; SKT, Syndrom-Kurz test; PO, Per os; P, piracetam group; C, control group; CABG, coronary artery bypass grafting.
